# Hospital bed supply and inequality as determinants of maternal mortality in China between 2004 and 2016

**DOI:** 10.1186/s12939-021-01391-9

**Published:** 2021-01-30

**Authors:** Fan Tian, Jay Pan

**Affiliations:** 1 HEOA Group, West China School of Public Health and West China Fourth Hospital, Sichuan University, No. 17, Section 3, Ren Min Nan Road, Chengdu, 610041 Sichuan China; 2 Institute for Healthy Cities and West China Research Center for Rural Health Development, Sichuan University, No. 17, Section 3, Ren Min Nan Road, Chengdu, 610041 Sichuan China

**Keywords:** Hospital beds, Inequality, Geographical distribution, Maternal mortality ratio, China

## Abstract

**Background:**

Driven by the government’s firm commitment to promoting maternal health, maternal mortality ratio (MMR) in China has achieved a remarkable reduction over the past 25 years. Paralleled with the decline of MMR has been the expansion of hospital bed supply as well as substantial reduction in hospital bed distribution inequalities, which were thought to be significant contributors to the reduction in MMR. However, evidences on the impact of hospital bed supply as well as how its distribution inequality has affected MMR remains scarce in China. Addressing this uncertainty is essential to understand whether efforts made on the expansion of healthcare resource supply as well as on improving its distribution inequality from a geographical perspective has the potential to produce measurable population health improvements.

**Methods:**

Panel data of 31 provinces in China between 2004 and 2016 were extracted from the national statistical data, including China Statistical Yearbooks, China Health Statistical Yearbooks and other national publications. We firstly described the changes in hospital bed density as well as its distribution inequality from a geographical perspective. Then, a linear mixed model was employed to evaluate the impact of hospital bed supply as well as its distribution inequality on MMR at the provincial level.

**Results:**

The MMR decreased substantially from 48.3 to 19.9 deaths per 100,000 live births between 2004 and 2016. The average hospital bed density increased from 2.28 per 1000 population in 2004 to 4.54 per 1000 population in 2016, with the average Gini coefficient reducing from 0.32 to 0.25. As indicated by the adjusted mixed-effects regressions, hospital bed density had a negative association with MMR (*β* = − 0.112, 95% *CI*: − 0.210--0.013) while every 0.1-unit reduction of Gini coefficient suggested 14.50% decline in MMR on average (*β* = 1.354, 95% *CI*: 0.123–2.584). Based on the mediation analysis, the association between hospital bed density or Gini coefficient with MMR was found to be significantly mediated by facility birth rate, especially during the period from 2004 to 2009.

**Conclusions:**

This study provided empirical evidences on China’s impressive success in the aspect of reducing MMR which could be attributed to the expansion of hospital beds as well as the improvement in its distribution inequality from a geographical perspective. Such findings were expected to provide evidence-based implications for long-term policy-making procedures in order to achieve rational healthcare resource allocations as well as promoting the equity and accessibility to obtaining health care from a holistic perspective. Constant efforts should be made on improving the equity in healthcare resource allocations in order to achieve the penetration of universal healthcare coverage.

**Supplementary Information:**

The online version contains supplementary material available at 10.1186/s12939-021-01391-9.

## Introduction

Maternal mortality ratio (MMR) is a globally recognized indicator which reflects the overall health of a population, the status of women in a society as well as the development status of the healthcare system. Since the Safe Motherhood Initiative was inaugurated in Kenya in 1987, many countries have focused on the reduction of the high burden of maternal health [1]. In 2000, a list of Millennium Development Goals (MDGs) was proposed by the United Nations, among which one of the critical goals to be achieved was a 75% reduction in the MMR between 1990 and 2015 [2]. Progress towards improving maternal health was found to be accelerated after that time point. According to the MDGs report, a 45% reduction of the MMR in a worldwide range was found between 1990 and 2013, from 380 to 210 maternal deaths per 100,000 live births [3]. Quite a number of developing regions such as Southern Asia and Sub-Saharan Africa have made substantial improvements in promoting maternal health. Despite the progress that has been achieved so far, regional inequalities remains in maternal health conditions, with 94% of all maternal deaths reported in low and lower middle-income countries in 2017 [4]. Therefore, the reduction of maternal mortality should be addressed as a constant priority as part of the global health and development agenda, especially in developing countries.

Over the past 25 years, China has achieved impressive progress in the promotion of maternal and child health (MCH) given its daunting population size as well as the diversity of counties. The MMR decreased strikingly from 88.8 to 21.7 deaths per 100,000 live births between 1990 and 2014 [5], which indicated that MDG 5 has been successfully achieved in China. A list of factors was considered as potential contributors to having accelerated such achievement. Specifically, a series of explicit policies and programs proposed since 1995 were thought to have addressed the promotion of maternal health as a critical priority as part of the health agenda including the Law on Infant and Maternal Health, the National Plan of Action for Women, and MMR Reduction and Neonatal Tetanus Elimination Program [6–10]. In addition, the Chinese government’s long-term commitment to increasing investment on MCH has significantly improved the access to antenatal and obstetric delivery care among healthcare facilities [11]. Financial burdens induced by medical cost have also been alleviated via the adoption of relevant social health insurance programs as well as financial compensations for MCH care [12]. Meanwhile, a well-functioning three-tiered MCH network covering the county, township and village levels was established, which has provided an effective medical referral system for patients at high risks as well as facilitating communications among different healthcare facilities while serving as an extensive supervision system for MCH expert training purposes [13, 14]. Last but not least, the health information system across the country has been substantially improved, which has greatly enhanced the MCH in terms of both monitoring and reporting capacities [14, 15]. The factors contributing to the reduction of MMR are complex, but progress achieved in the expansion of healthcare resources including maternal healthcare facilities, hospital beds as well as healthcare professionals, as a foundation to ensure a high coverage of MCH care, was thought to be an essential contributor in this aspect [12, 14].

Since the initiation of the nationwide reform and opening-up process in 1978, China has made remarkable economic and social developments. As the result, constant efforts have been made at governmental levels on increasing financial investments in healthcare systems, especially after the outbreak of Severe Acute Respiratory Syndrome (SARS) in 2003, which induced a remarkable growth in China’s healthcare resources. Between 2004 and 2016, the number of health facilities increased from 849,140 to 983,394 and the number of hospital beds per 1000 population increased from 2.51 to 5.37 [5]. Paralleled with the expansion of healthcare facilities was the expansion of healthcare workforce, with the number of healthcare personnel per 1000 population increasing from 3.53 to 6.12 [5].

Despite such remarkable progress, constantly raising inequalities embedded in the geographic distribution of healthcare resources remains a critical problem to be concerned [16, 17]. In 2005, doctor density in urban areas was reported more than twice that in rural areas, with nurse density showing more than a three-fold disparity, which presented a strong urban bias in the allocation of healthcare resources [18]. Similar disparity could be found in the hospital bed density at the regional level [16]. In attempt to tackle with this critical issue, Chinese government has included a main focus on improving the reasonable distribution of healthcare resources and promoting equity of healthcare [19]. In spite of the healthcare inequalities constantly embedded among different regions and provinces in China, such gaps were found to be narrowed over time. For example, between 2004 and 2016, the Gini coefficients of hospital bed density between provinces decreased from 0.129 to 0.071 [5], thus indicating a substantial reduction of healthcare resource inequalities.

From the perspective of suppliers’ side, healthcare resources are thought to serve as the prerequisites for improving accessibility to healthcare, while equalities embedded in geographic distributions of healthcare resources are believed as the key to timely access to healthcare services. It has been indicated by empirical evidences that inequities embedded in healthcare resource and service distributions would significantly exacerbate the disparities of healthcare outcomes as well as the quality of lives [18, 20, 21]. However, to our knowledge, accurate information as to the impact of changes in healthcare resource supply as well as how its distribution inequality has affected MMR in China remains scarce based on previous literature. As the result, it has become quite essential to understand whether efforts made on the expansion of healthcare resource supply as well as on reducing its inequality in geographic distributions have the potential to produce measurable population health promotion. Based on these considerations, in this study hospital beds was adopted as the proxy indicator of healthcare resources, which was aimed at investigating the impact of healthcare resource supply as well as its distribution inequality on MMR among 31 provinces in China between 2004 and 2016.

## Hypothesis

As the core building blocks of healthcare systems, healthcare resources are widely recognized as critical determinants of population healthcare outcomes. Like all the other productive processes in the field of economics, healthcare resources can be regarded as the input while population healthcare outcomes as the output in a healthcare system. Based on such considerations, it would be reasonable to assume that healthcare resources have a diminishing return on population health while controlling for all the other health-related factors. That is, an additional rise in healthcare resources would create sustained improvement in population health, while such improvement would be conversely diminished along with higher levels of healthcare resources. In the field of economics, the law of diminishing marginal productivity has been widely adopted as a common rule in production theory [22], and also extensively used in identifying social determinants of health, especially in the aspect of investigating the negative impact of social income inequality on population health [23–25].

**Fig. 1:**
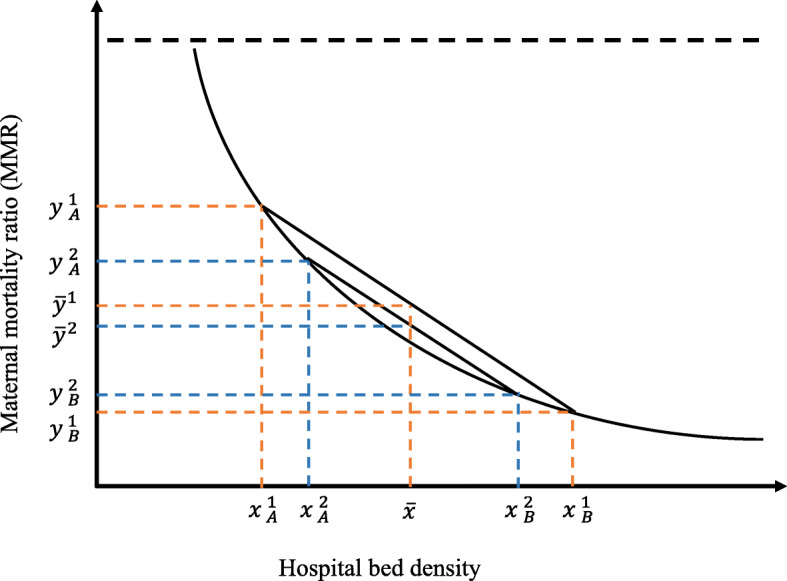
Convex relationship between hospital bed density and MMR

Figure 1 depicts the relationship between healthcare resources and population healthcare outcomes. The x-axis refers to healthcare resources, which is illustrated by hospital bed density in this study, and y-axis denotes the maternal mortality ratio (MMR). A convex relationship between hospital bed density and MMR was depicted in this figure showing two facts: (1) Increased hospital bed provision and lower MMR will be observed when other factors associated with MMR remain unchanged. (2) The reduction in MMR induced by a certain raise of hospital bed density will be gradually diminished with constantly increased hospital bed density.

Now we turn to the potential effect of geographic distributions of hospital bed density on MMR. To be concise, we supposed two counties (A and B) in a hypothetical province, where two scenarios (1 and 2) of interests were embedded for analysis.

### Scenario 1

County A had a lower hospital bed density (${x}_A^1$) compared with county B (${x}_B^1$), which resulted in a higher MMR (${y}_A^1$) in county A in contrast with county B (${y}_B^1$). The average hospital bed density in the province for scenario 1 was (${x}_A^1+{x}_B^1$)/2, which was shown as $\overline{x}$ in Fig. 1, while the average MMR was (${y}_A^1+{y}_B^1$)/2, shown as ${\overline{y}}^1$.

### Scenario 2

We narrowed the gap in hospital bed density between county A and B while keeping the average hospital bed density ($\overline{x}$) as a constant value. Hospital beds in county A increased to ${x}_A^2$ and that in county B decreased to ${x}_B^2$. Consequently, we could observe a substantial reduction in MMR of county A (${y}_A^2$), while a slight rise in that of county B (${y}_B^2$) was identified. The averaged hospital bed density remained $\overline{x}$, while the average MMR changed to ${\overline{y}}^2$, equaling to (${y}_A^2+{y}_B^2$)/2.

Comparing scenario 1 with 2, a significant reduction of average MMR could be observed from ${\overline{y}}^1$ to ${\overline{y}}^2$, indicating that the reduction of MMR in county A has greatly offset the raise of MMR in county B. Based on the static comparison of the two theoretical scenarios, it could be concluded that improved equality in the distribution of hospital beds density between the two counties would very much likely to achieve promoted healthcare outcomes in the province. As the result, such hypothesis was proposed and adopted for the empirical analysis that both the expansion of hospital bed supply and the improvement in its geographic distributions would achieve in a reduction of average MMR.

## Methods

### Data sources

Our main analysis was conducted at the provincial level. The MMRs in 31 provinces between 2004 and 2016 were retrieved from the National Health Statistical Yearbooks [5]. The reported MMRs in the yearbooks were annually collected by the National Maternal Mortality Surveillance System, a well-developed population-based maternal death registry system which was established by the Ministry of Health of China in 1989 [11]. The accuracy of the data was guaranteed by a rigorous quality control mechanism including the standardized data collection procedures, strict data review and data management [26].

As the purpose of our study was to evaluate the impact of hospital bed supply as well as its distribution inequality on MMR, three data resources were adopted for extracting hospital bed data. In the first step, provincial-level data on hospital beds between 2004 and 2016 was directly obtained from National Health Statistical Yearbooks [5]. In order to measure the inequality of hospital bed distribution within each province, we further extracted county/city level data on hospital beds from China County/City Social and Economic Statistical Yearbooks [27, 28]. According to the divisions of administrative areas in China in 2015 [29], there was a total of 34 provinces with 2850 county level administrative units. Due to the data availability, we totally obtained hospital bed data on 2347 counties/districts (2082 counties in rural areas and 265 districts in urban areas) among 31 provinces in China, except for Hong Kong Special Administrative Region (SAR), Macao SAR and Taiwan province. We utilized the variations in hospital beds among the 2347 counties/districts to calculate the provincial inequality index of geographic distributions of healthcare resources.

Additionally, facility birth rate was extracted from the National Health Statistical Yearbooks [5]. Other social-economic characteristics of each province were collected from the China Statistical Yearbook [30] Detailed information for each variable were presented in Additional file 1: Appendix Table 1.

### Outcome

The key outcome variable is maternal mortality ratio (MMR), which is measured by the annual number of maternal deaths per 100,000 live births for a specified time period. Maternal deaths are defined as women who died from any cause related to or aggravated by pregnancy or its management except for accidental or incidental causes during pregnancy and childbirth or within 42 days of termination of pregnancy, irrespective of the duration and site of the pregnancy [31].

### Independent variables

Hospital bed density and its inequality in geographic distribution were two primary independent variables in our study. According to China Health Statistical Yearbooks, hospital bed density was defined as the total number of beds in healthcare institutions by the end of Dec 31st, per 1000 registered population in each region [5]. The hospital beds included regular beds, care beds and makeshift beds, but excluded the pre-delivery beds, beds in outpatient observation rooms and beds for newborn babies in obstetric wards [5]. According to the definition, the reported hospital beds mainly refer to the beds that are available or potentially available for health professionals to provide medical care for patients. In China’s healthcare delivery system, the pre-delivery beds are solely used to help the pregnant women prepare for the deliveries, which are different from obstetric beds where obstetricians actually provide delivery services. Thus, the pre-delivery beds were excluded from the counts of hospital beds.

The inequality in geographic distribution of hospital beds within each province was measured by Gini coefficient, which was one of the most widely used indicators for describing social and economic conditions [32–34]. The Gini coefficient was also recommended as a summarized measure of inequalities in health and has been applied in numerous empirical studies [16, 20, 35]. In this study, we employed the geometric approach to define this index, with a calculation formula defined as follows:

1 $$ Gini=\frac{n+1}{n}-\frac{2}{n^2{\mu}_y}\sum \limits_{i=1}^n\left(n+1-i\right){y}_i $$

where *n* is the total number of counties within a province, *μ*_*y*_ is the mean of hospital bed density among all counties, and *y*_*i*_ refers to the county *i* ’s hospital bed density. When calculated, counties should be ranked in ascending order of *y*_*i*_ at first and corresponding population numbers are usually considered as survey weights in the equation. The value of the Gini coefficient ranges from 0 and 1, with higher values indicating higher inequality in geographic distribution of hospital bed [36]. According to previous studies, a Gini coefficient smaller than 0.2 means low inequality level, and values higher than 0.4 indicates extreme inequality [37, 38].

The following variables that may confound the association between hospital bed supply as well as its inequality in geographic distributions and MMR were considered as covariates in our analyses: crude birth rate (‰), female illiteracy (i.e., the proportion of women aged 15 years or older who were illiterate) (%), gross domestic product (GDP) per capita (1000 Yuan), and urbanization rate (percentage of urban population) (%). The GDP per capita was adjusted for inflation based on the Consumer Price Index (CPI) (National Bureau of Statistics, 2017). We also included facility birth rate (%), as a crucial indicator of maternal healthcare utilization, when exploring the casual path from hospital bed density and Gini coefficient to MMR.

### Statistical analysis

First, we performed descriptive analysis to investigate the time trends and regional variations in MMR between 2004 and 2016. Based on GDP per capita [30], 31 provinces were categorized into five groups: the highest, upper middle, middle, lower middle, and lowest income. Line plot and box plot were used for depicting the time series and regional disparities in MMR in each province by year and income level.

Second, we depicted the time trends and regional variations in hospital bed density as well as its distribution inequality between 2004 and 2016. Two different methods were considered for measuring hospital bed density. The first method was to divide the counties into six categories based on hospital beds per 1000 population, and different shading indicated varied changes in hospital bed density. From the central governments’ definition, China was divided into three regions (eastern, central and western region) based on the disparities embedded in economic progress. Moreover, large variations were found in population quantity and land size at the county level between the eastern and western regions, where a vast territory with a sparse population was typically identified in western counties. Due to small population and large land size, the values of hospital bed density in western counties might conceal the real pattern of geographic distribution in hospital beds. Thus, the second approach further adjusted the land size of each county when calculating the bed density. In addition, we used box plot and bar plot to describe the time trends and variations in inequalities of hospital bed distributions (measured by Gini coefficient) by year and province.

Third, we used a linear mixed model to examine the effect of changes in hospital bed supply as well as its distribution inequality on MMR between 2004 and 2016. Since the correlated structure of yearly reported MMR within each province could be incorporated for analysis into the mixed model, both within- and between-province components of variation in MMR could be distinguished. Moreover, an autoregressive covariance structure was fitted for residual effects to account for serial correlation in MMR across time within the provinces. The model was set as follows:

2 $$ {\displaystyle \begin{array}{c}\log \left({MMR}_{ij}\right)={\alpha}_{0j}+{\beta}_1{Bed}_{ij}+{\beta}_2{Gini}_{ij}+{\mathbf{X}}_{\boldsymbol{ij}}^{\prime}\lambda +{\varepsilon}_{0 ij}\\ {}{\alpha}_{0j}={\alpha}_0+{\mu}_{0j}\\ {}{u}_{0j}\sim N\left(0,{\sigma}_{\mu_0}^2\right)\\ {}{\varepsilon}_{0 ij}\sim N\left(0,{\sigma}_{\varepsilon_0}^2\right)\end{array}} $$

where *i* denoted the year ranged from 2004 to 2016 and *j* indexed the provinces. The *MMR*_*ij*_ was the maternal mortality ratio for province *j* in year *i*. *Bed*_*ij*_ and *Gini*_*ij*_ were two variables of interest, which indicated the hospital bed density and Gini coefficient for province *j* in year *i*. **X**′_***ij***_ denoted a vector of provincial characteristics, including female illiteracy, crude birth rate, GDP per capita and percentage of urban population. Year dummy variables were also included in the regression model. To address the positive skewed distribution of *MMR*_*ij*_, we employed the natural logarithm of this ratio to fulfill the assumption of linearity. Thus, the intercept *α*_0*j*_ indicated the average logarithm of MMR between 31 provinces, equaling *α*_0_ (total mean of log(*MMR*_*ij*_)) plus a random effects’ terms *μ*_0*j*_. *ε*_0*ij*_ was the error term. The coefficient on *Bed*_*ij*_ (*β*_1_) and *Gini*_*ij*_ (*β*_2_) were two parameters of primary interest. As we used logarithm of MMR in the model, a one-unit increase in the estimated coefficients $\hat{\beta}$ would produce an expected increase in log(*MMR*_*ij*_) of $\hat{\beta}$ units. In terms of *MMR*_*ij*_ itself, this meant that the expected value of *MMR*_*ij*_ was multiplied by ${e}^{\hat{\beta}}$. Thus, we exponentiated the coefficient $\hat{\beta}$, subtracted one from this number, and multiplied by 100, which gave a corresponding percent increase (or decrease) in the *MMR*_*ij*_ for every one-unit increase in the independent variables. So given a negative value of estimated coefficient ${\hat{\beta}}_1$, one-unit increase in hospital bed density was associated with a $\left({e}^{{\hat{\beta}}_1}-1\right)\times 100$ percent decline in the MMR. For a positive value of ${\hat{\beta}}_2$, a 0.1-unit increase in Gini coefficient, indicating a relatively larger inequality in geographic distribution of hospital bed, was associated with a $\left({e}^{{\hat{\beta}}_2\times 0.1}-1\right)\times 100$ percent increase in the MMR.

Fourth, we explored whether the effect of hospital bed density and Gini coefficient on MMR was mediated by facility birth rate as well as the extent to which this effect was mediated. The three steps approach outlined by Baron and Kenny [39] was used to assess the mediating effect. Three mixed-effects regression models were estimated: (1) a model examining the effect of hospital bed density (or Gini coefficient) on MMR; (2) a model examining the effect of hospital bed density (or Gini coefficient) on facility birth rate; (3) a model for MMR conditioning on hospital bed density (or Gini coefficient) and facility birth rate. The mediating influence of facility birth rate on the effect of hospital bed density (or Gini coefficient) on MMR was tested using the Sobel test [40]. Moreover, we performed the mediation analysis for two periods respectively (2004 to 2009 and 2010 to 2016). Since the maternal mortality reduction and neonatal tetanus elimination programme was launched in 1999 [10], facility birth rate in China has been steadily increased. In 2009, this program was implemented in a nationwide range in order to provide free hospital delivery for all women in China [41], which achieved universal facility births by 2009, with an average rate of 94.51% reported in 31 provinces (Table 1). Thus, we conducted the mediation analysis for two different periods to examine the mediating role of facility birth rate. In addition, it should be noted that there are some limitations in this traditional mediation analysis implemented within the framework of linear structural equation models [42]. A new approach, namely causal mediation analysis, has been developed under the counterfactual framework [42]. But in this analysis, we primarily aims to identify the role of facility birth rate on the association between hospital bed density (or Gini coefficient) and MMR, instead of identifying the causality which needs more assumptions to be clarified. In this regards, the traditional mediation method was adopted in the analysis.

**Table 1:** Characteristics of 31 provinces in China, 2004–2016

Characteristics	Between-province variations						Within-province change, 2004 to 2016
	Mean (95% ***CI***)			Median (Quantile range)			
	2004	2009	2016	2004	2009	2016	
**Independent Variables**							
Hospital beds per 1000 population	2.76 (2.44, 3.08)	3.48 (3.22, 3.73)	5.36 (5.11, 5.61)	2.41 (0.92)	3.31 (0.84)	5.39 (1.27)	2.60 (2.22, 2.97)
Gini coefficient	0.32 (0.28, 0.35)	0.30 (0.26, 0.33)	0.25 (0.22, 0.28)	0.34 (0.09)	0.31 (0.09)	0.26 (0.07)	−0.07 (−0.09, − 0.05)
Birth rate, ‰	11.45 (10.26, 12.65)	11.38 (10.40, 12.36)	11.80 (10.72, 12.88)	11.67 (4.56)	11.70 (4.17)	12.18 (3.93)	0.35 (−0.36, 1.06)
Female illiteracy, %	16.18 (12.67, 19.69)	11.84 (8.75, 14.93)	9.17 (6.11, 12.22)	14.22 (12.03)	9.90 (7.17)	7.32 (5.12)	−7.01 (−8.25, −5.77)
GDP per capita, 1000 yuan	19.39 (14.01, 24.76)	34.75 (27.25, 42.24)	56.77 (47.33, 66.20)	13.23 (12.45)	26.33 (22.44)	46.38 (32.06)	37.38 (32.13, 42.63)
Urbanization rate, %	29.94 (24.02, 35.86)	49.27 (43.89, 54.65)	57.85 (53.28, 62.42)	25.99 (12.47)	46.00 (15.65)	56.21 (14.39)	27.91 (24.23, 31.59)
Facility birth rate, %	81.68 (75.40, 87.97)	94.91 (91.38, 98.44)	99.47 (98.90, 100.04)	85.40 (21.40)	98.50 (3.80)	100 (0.40)	17.79 (11.89, 23.69)
**Dependent Variable**							
Maternal mortality ratio, per 100,000 live births	55.00 (34.56, 75.44)	28.84 (14.33, 43.35)	16.63 (9.80, 23.46)	42.80 (43.70)	19.20 (16.40)	12.70 (8.10)	−38.37 (−52.46, −24.28)

Note: *CI*: confidence interval

All analyses were performed using ArcGIS 10.5, SAS 9.4, and R 3.4.3.

## Results

### Time trends and regional variations in MMR, China, 2004–2016

**Fig. 2:**
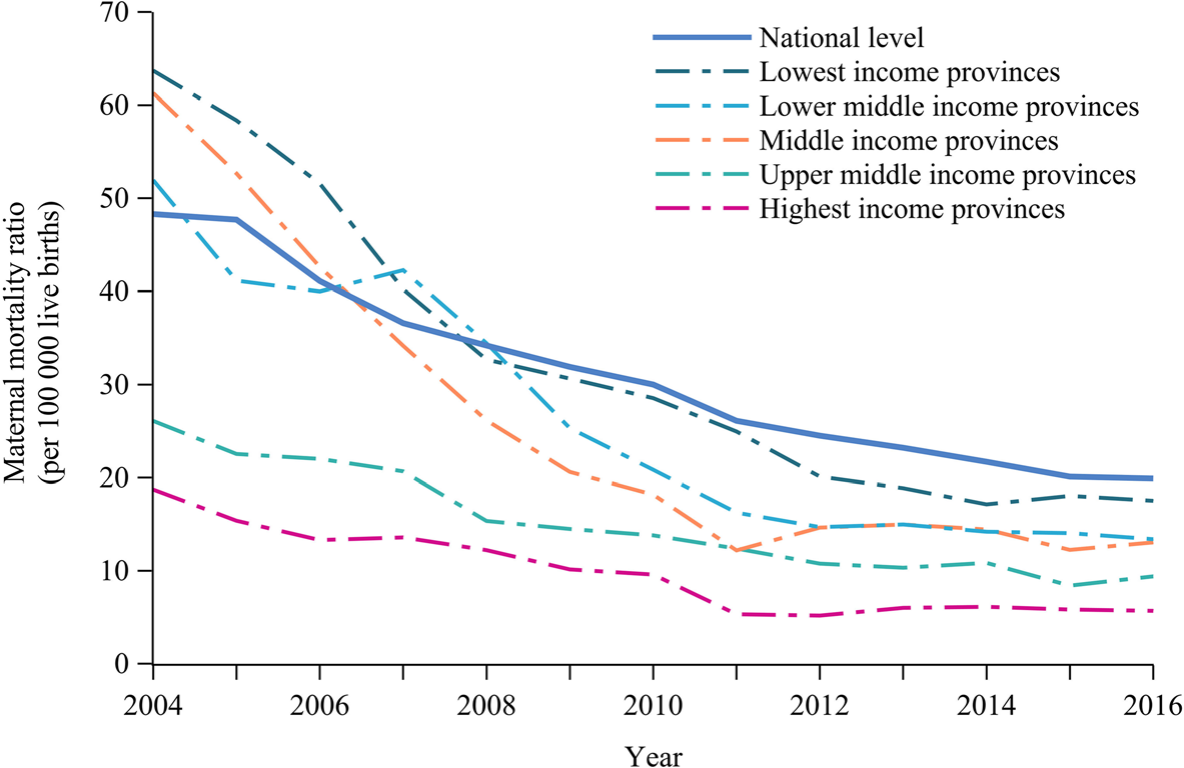
Maternal mortality ratio by year and income level of provinces, China, 2004–2016

The overall MMR decreased substantially from 48.3 deaths per 100,000 live births in 2004 to 19.9 deaths per 100,000 live births in 2016 (Fig. 2). The patterns of reduction in MMR could also be found in provinces at different income levels. For example, MMR in provinces at the lowest income levels reduced from 63.7 to 17.5 deaths per 100,000 live births between 2004 and 2016. A dramatic reduction was also identified in middle-income provinces, with the MMR decreasing from 61.3 to 13.1 deaths per 100,000 live births. In addition to the drastically accelerated reduction in the overall MMR, gaps between provinces with different socioeconomic development status were also substantially narrowed (Fig. 2 and Additional file 1: Appendix Table 2). The absolute difference in MMR between the highest and the lowest income provinces declined from 45.0 to 11.9 deaths per 100,000 live births between 2004 and 2016.

**Fig. 3:**
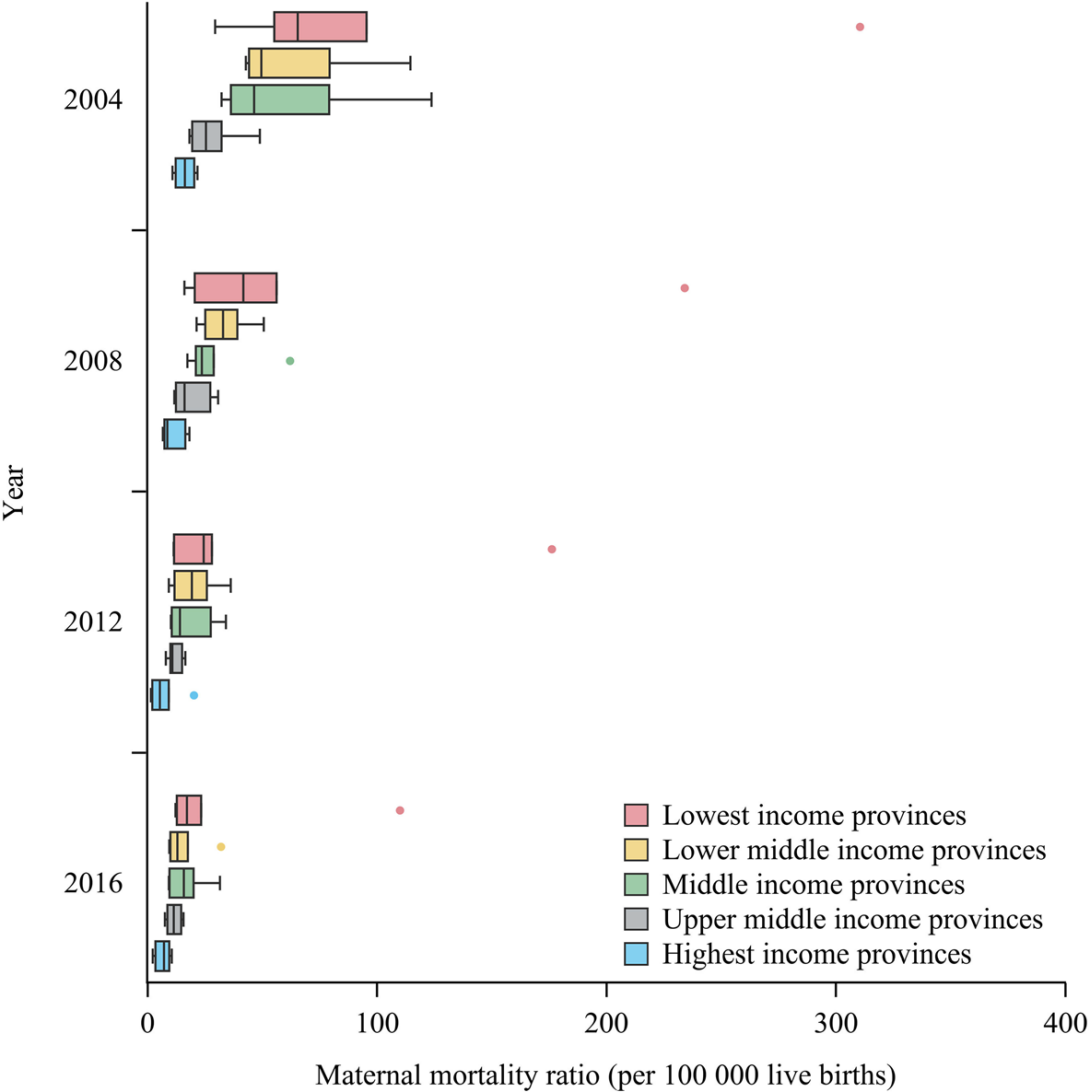
Regional variations in maternal mortality ratio, China, 2004–2016

Little variation was found in MMR across provinces among the highest and upper middle-income levels, while large variations were found within the middle, lower middle- and lowest-income provinces in terms of mortality (Fig. 3). The greatest variation was found in the lowest income provinces, where MMR ranged from 310.4 to 10.8 deaths per 100,000 live births in 2004. Although such large variations have become smaller from 2004 onwards, it should be noted that Tibet, as the largest outlier in every reported year, was still lagging behind.

### Time trends and regional variations in hospital bed density and inequality, China, 2004–2016

**Fig. 4:**
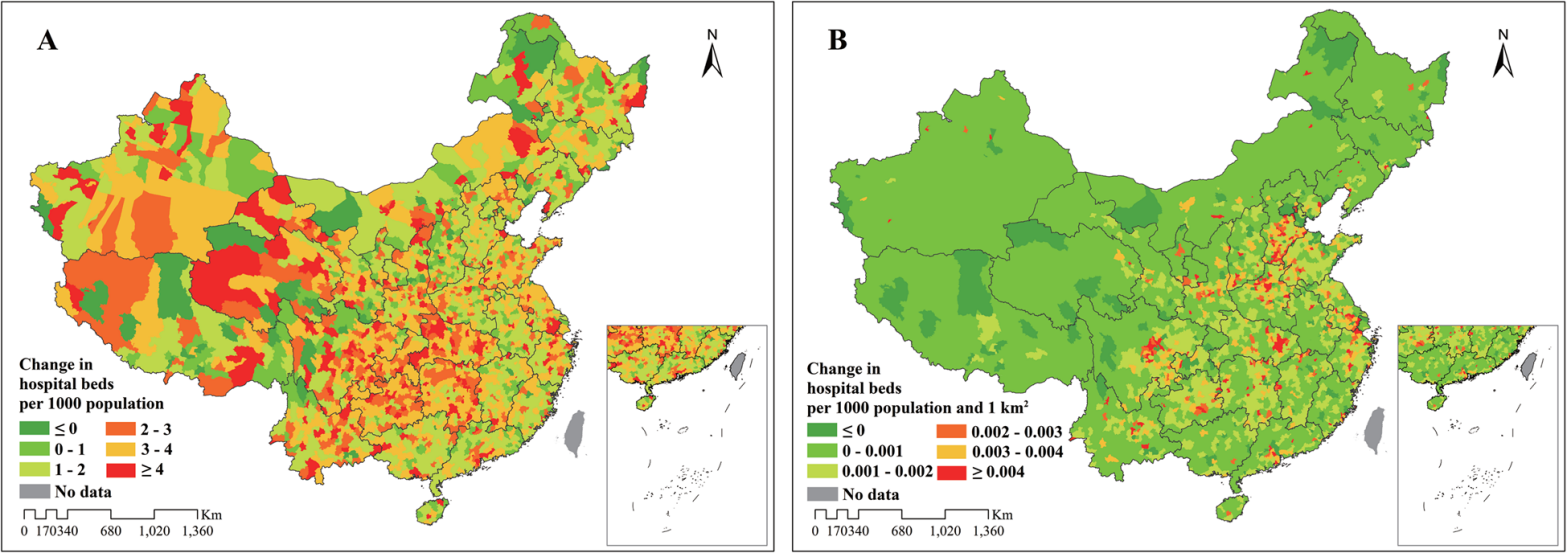
Changes in geographic distribution of hospital beds by county, China, 2004–2016

Paralleled with the decline in MMR has been the huge raise in hospital bed supply. Specifically, the total number of hospital beds increased from 3,268,374 in 2004 to 7,410,453 in 2016, corresponding to 2.53 per 1000 population in 2004 and 5.37 per 1000 population in 2016, respectively. At the county level, average hospital bed supply increased from 2.28 per 1000 population (95% *CI*: 2.21–2.34) to 4.54 (95% *CI*: 4.45–4.64) per 1000 population between 2004 and 2016 (Fig. 4a). Compared with the eastern region, counties in western and central regions demonstrated a larger expansion in hospital bed on average, with a raise of 2.45 and 2.22 per 1000 population versus 2.05 per 1000 population in the eastern region. However, after we further adjusted the county area in the process of measuring the hospital bed density, such trend previously found in the changes of hospital bed supply was reversed (Fig. 4b). Western counties were found to present a much lower growth in hospital bed quantities compared with more developed eastern counties, with an increase of 0.0011 to 0.0014 hospital beds per 1000 population per km^2^ (Additional file 1: Appendix Table 3). Additionally, as the result of having smaller population sizes, western counties appeared to have larger variations in hospital bed density compared with eastern counties. The absolute changes in western counties ranged from a loss of 10.78 to a gain of 14.70 hospital beds, with a median gain of 2.27 bed per county. In contrast, hospital bed changes in eastern counties varied from a loss of 2.36 to a gain of 11.50 hospital beds, with a median gain of 1.89 bed per county. The trends of variations between different regions were also reversed when using hospital beds per 1000 population and 1 km^2^ as the units for measurement. Despite a list of remarkable progress achieved in the expansion of hospital bed supply, some counties in the western region still demonstrated negative changes in this aspect as the result of disproportionate removal of hospital beds as well as the increase of the general population size.

**Fig. 5:**
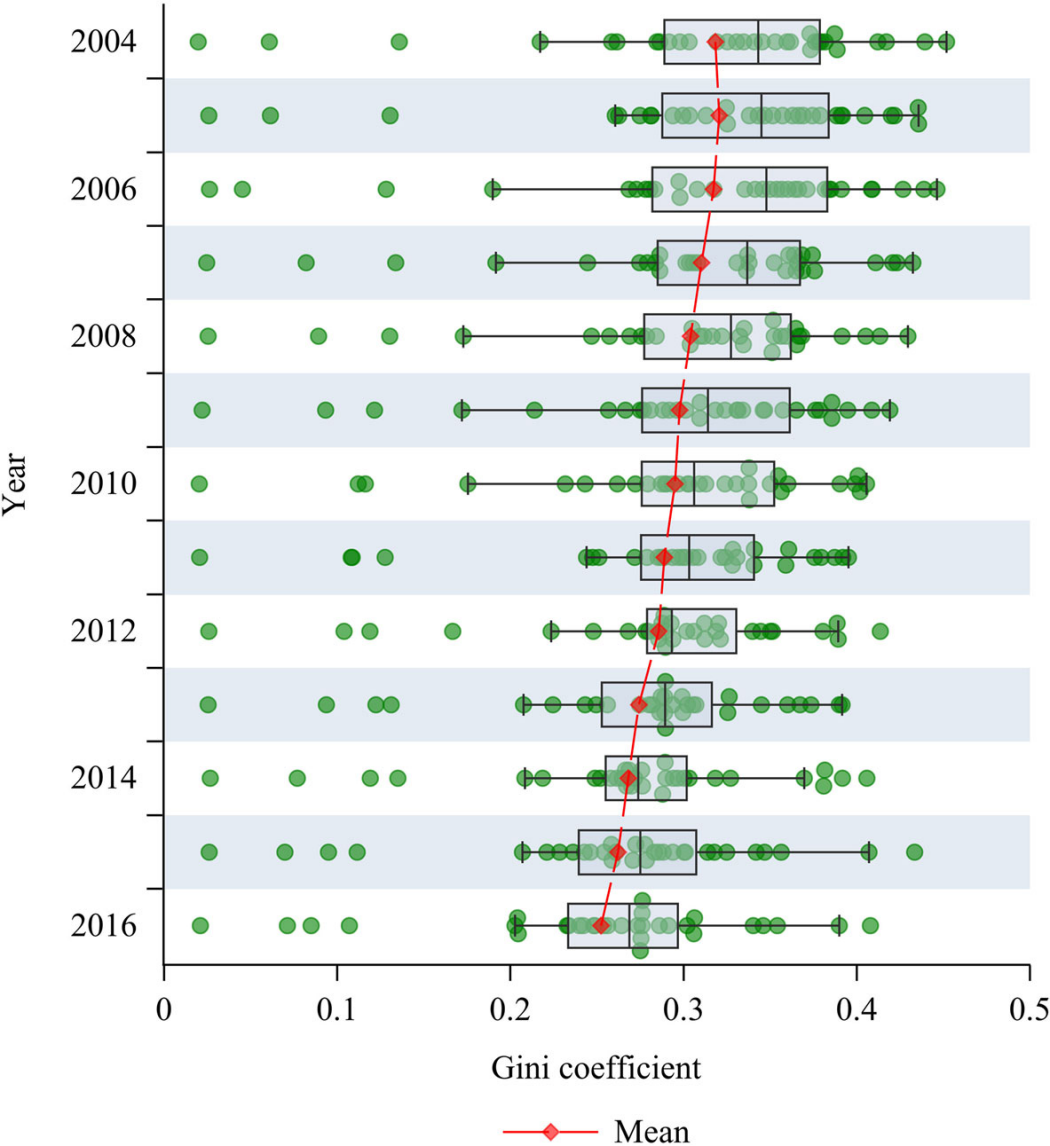
Overall inequalities in hospital bed distribution in China, 2004–2016

**Fig. 6:**
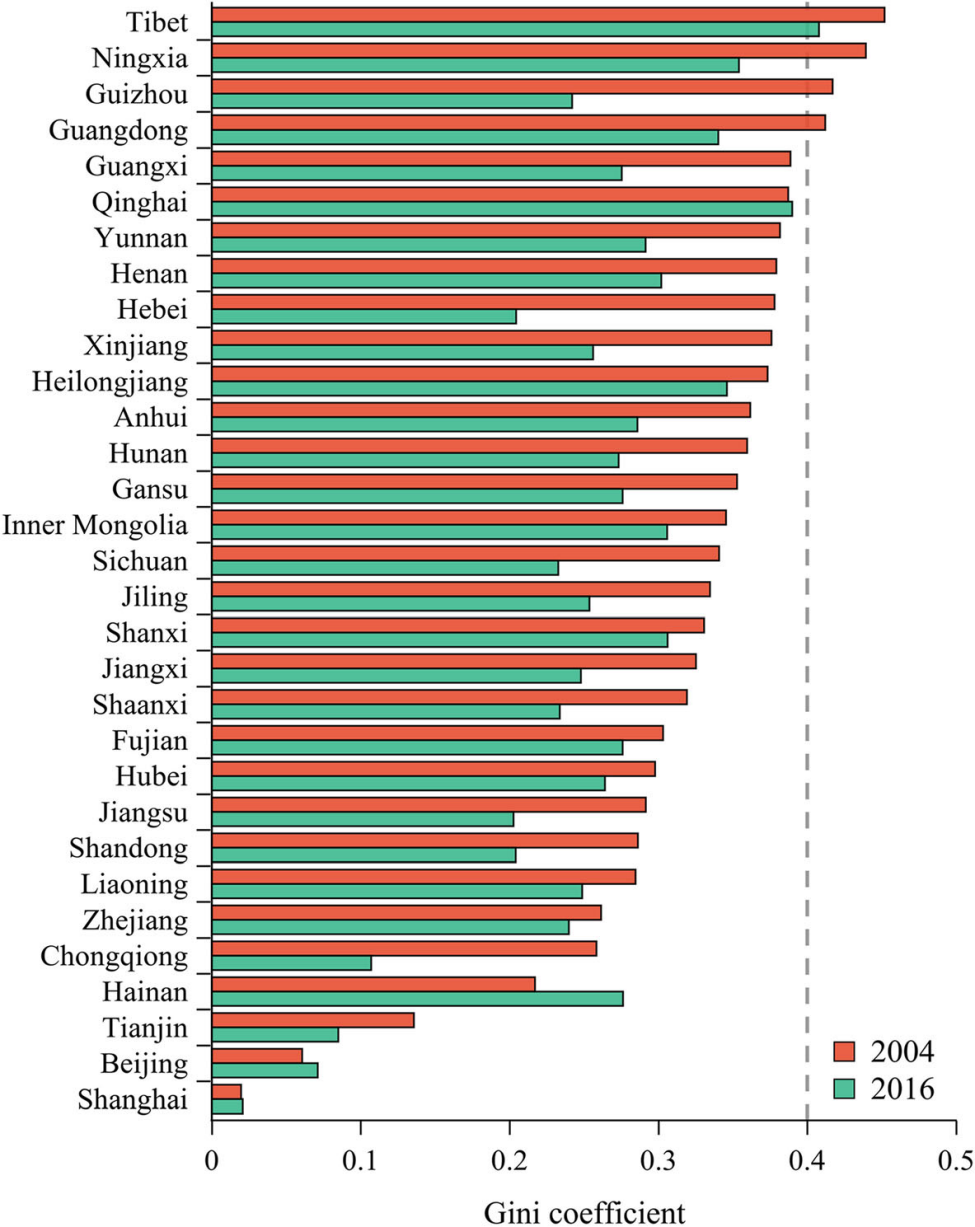
Inequalities in hospital bed distribution by province, China, 2004–2016

During the past 13 years, the overall inequalities in geographic distribution of hospital beds have been improved steadily in China (Fig. 5). The average Gini coefficient declined from 0.32 (95% *CI*: 0.28–0.36) in 2004 to 0.25 (95% *CI*: 0.22–0.28) in 2016 in spite of a slighter increase of 0.002 between 2004 and 2005. Most of the provinces were found to have achieved a more equal resource distribution of hospital beds within each province from 2004 onwards (Fig. 6). For example, Guizhou province in western China has made the largest improvement in hospital bed distribution inequality, with its regional Gini coefficient reduced from 0.42 in 2004 to 0.24 in 2016. The Gini coefficient in three provinces (Ningxia, Guizhou and Guangdong) which were all reported greater than 0.4 in 2014, all dropped below 0.4 in 2016. Overall, the absolute changes of Gini coefficient in 31 provinces varied from a reduction of 0.18 (Guizhou province) to a raise of 0.06 (Hainan province), with a median decrease of 0.08 per province. It should be noted that four provinces including Hainan, Beijing, Qinghai and Shanghai presented a reversed trend in Gini coefficient between 2004 and 2016. Moreover, such variations of inequalities in hospital bed distributions still remained among different provinces in spite of considerable narrowing of such gaps over the past decade (Figs. 5 and 6).

### Associations between hospital bed density and inequality and MMR

Table 1 summarized the socio-economic characteristics and maternal healthcare utilization indicator of each province by the mean with 95% confidence intervals (*CI*s) and median with quantile range. Changes between 2004 and 2016 of these variables were also reported.

Before using the mixed-effects regression model, we tested the collinearity of included independent variables and then excluded the urbanization rate from the regression model due to its high correlation with GDP per capita (*r* = 0.876) and the largest variance inflation factor (*VIF* = 6.519) (Additional file 1: Appendix Table 4 and 5). In the mixed-effects regression model, we firstly investigated whether there was a significant difference in MMR among different provinces, and to which extent such provincial level variations could be quantified by clustering this ratio across time within provinces. Then, we expanded the empty model (model 1) by including hospital bed density, Gini coefficient, year dummy variables, socio-economic factors and facility birth rate step by step for examining the robust of the estimates.

Table 2 provided the estimates of the mixed-effects regression model, where the dependent variable was the natural logarithm of MMR. The marginal effects were reported, with the corresponding 95% confidence interval (*CI*). The null model with no predictors (model 1) showed a significant variation in MMR between provinces (${\sigma}_{\mu_0}^2$ = 0.506). The intraclass correlation (*ICC*) was utilized to measure the proportion of total variance in MMR that can be explained at the provincial level. There was a large *ICC* in model 1 (*ICC* = 0.679), suggesting the provincial effects on MMR cannot be neglected. In model 2, the estimated coefficient for hospital bed per 1000 population was − 0.201 and statistically different from zero at the 5% significance level, which indicated that the MMR would be reduced by 18.21% on average with a raise of 1 hospital bed per 1000 population. Moreover, the Gini coefficient was found to have a positive association with MMR (*β* = 2.036, 95% *CI*: 0.941–3.131), indicating that a more equitable geographical distribution of hospital bed density was associated with a lower MMR. Year dummy variables were also included in model 2. We found an increase of *ICC* and a huge reduction of − 2 residual log likelihood in model 2, which implied this model was preferable compared with the empty model.

**Table 2:** Mixed-effects regression models of the association of hospital bed supply and inequality with maternal mortality ratio, China, 2004–2016

Variables	Log of maternal mortality ratio				
	Model 1	Model 2	Model 3	Model 4	Model 5^d^
**Fixed effects,** ***β*** **(95%** ***CI*)**					
Hospital beds per 1000 population		−0.201^c^ (−0.268, −0.134)	−0.178^c^ (−0.245,-0.110)	−0.112^c^ (− 0.191,-0.033)	−0.112^b^ (− 0.210, − 0.013)
Gini coefficient		2.036^c^ (0.941, 3.131)	1.931^c^ (0.840, 3.023)	1.837^c^ (0.762, 2.911)	1.354^b^ (0.123, 2.584)
Birth rate, ‰			−0.005 (− 0.035, 0.026)	−0.010 (− 0.041, 0.020)	−0.012 (− 0.046, 0.023)
Female illiteracy, %			0.020^c^ (0.008, 0.031)	0.018^c^ (0.006, 0.030)	0.022^c^ (0.009, 0.035)
Log of GDP per capita				−0.382^c^ (− 0.645, − 0.118)	−0.406^c^ (− 0.710, − 0.101)
Year	No	Yes	Yes	Yes	Yes
**Random effects, variance (*SE*)**					
Variance between provinces	0.506 (0.135)	0.405 (0.108)	0.319 (0.089)	0.243 (0.071)	0.222 (0.071)
Variance within provinces	0.239 (0.018)	0.055 (0.004)	0.055 (0.004)	0.055 (0.004)	0.062 (0.006)
Residual covariance					0.401 (0.058)
*ICC*	0.679	0.881	0.854	0.816	0.783
-2 Residual log likelihood	671.36	157.92	162.86	157.95	109.70
*AIC*	675.36	161.92	166.86	161.95	115.7

Note: *CI*: confidence interval, *SE*: standard error, *ICC*: intraclass correlation, *AIC*: Akaike info criterion. ^c^, ^b^ and ^a^ denote 1, 5 and 10% significance levels, respectively. ^d^, Compared with model 4, model 5 further took the serial covariance in MMR across time within provinces into account when measuring the residual effects

From model 3 to model 4, hospital bed density and Gini coefficient continued to demonstrate significant associations with MMR after adjusting a list of socio-economic factors (i.e., crude birth rate, female illiteracy, GDP per capita). Based on model 4, model 5 further employed a first-order autoregressive covariance structure to fit the residual effects as well as testing the model’s robustness. In model 5, hospital bed density still showed a negative association with MMR (*β* = − 0.112, 95% *CI*: − 0.210--0.013). The Gini coefficient was found to be significantly positively associated with MMR (*β* = 1.354, 95% *CI*: 0.123–2.584), which indicated that the MMR will be reduced by 14.50% on average with a 0.1 improvement of inequality distribution on hospital bed (0.1 unit decrease of Gini coefficient). It is noteworthy that the logarithm of GDP per capita presented a significant negative association with MMR (*β* = − 0.406, 95% *CI*: − 0.710--0.101), which provided a practical evidence for describing the contribution of economic development levels on the reduction of MMR at the provincial level. Additionally, the results reported that increased female illiteracy rate was significantly associated with higher MMR (*β* = 0.022, 95% *CI*: 0.009–0.035). Furthermore, the − 2 log likelihood values and Akaike information criterion (*AIC*) for the full adjusted model were both smaller compared with model 5, which revealed that the final model had a better model fit.

### Mediation analysis

**Fig. 7:**
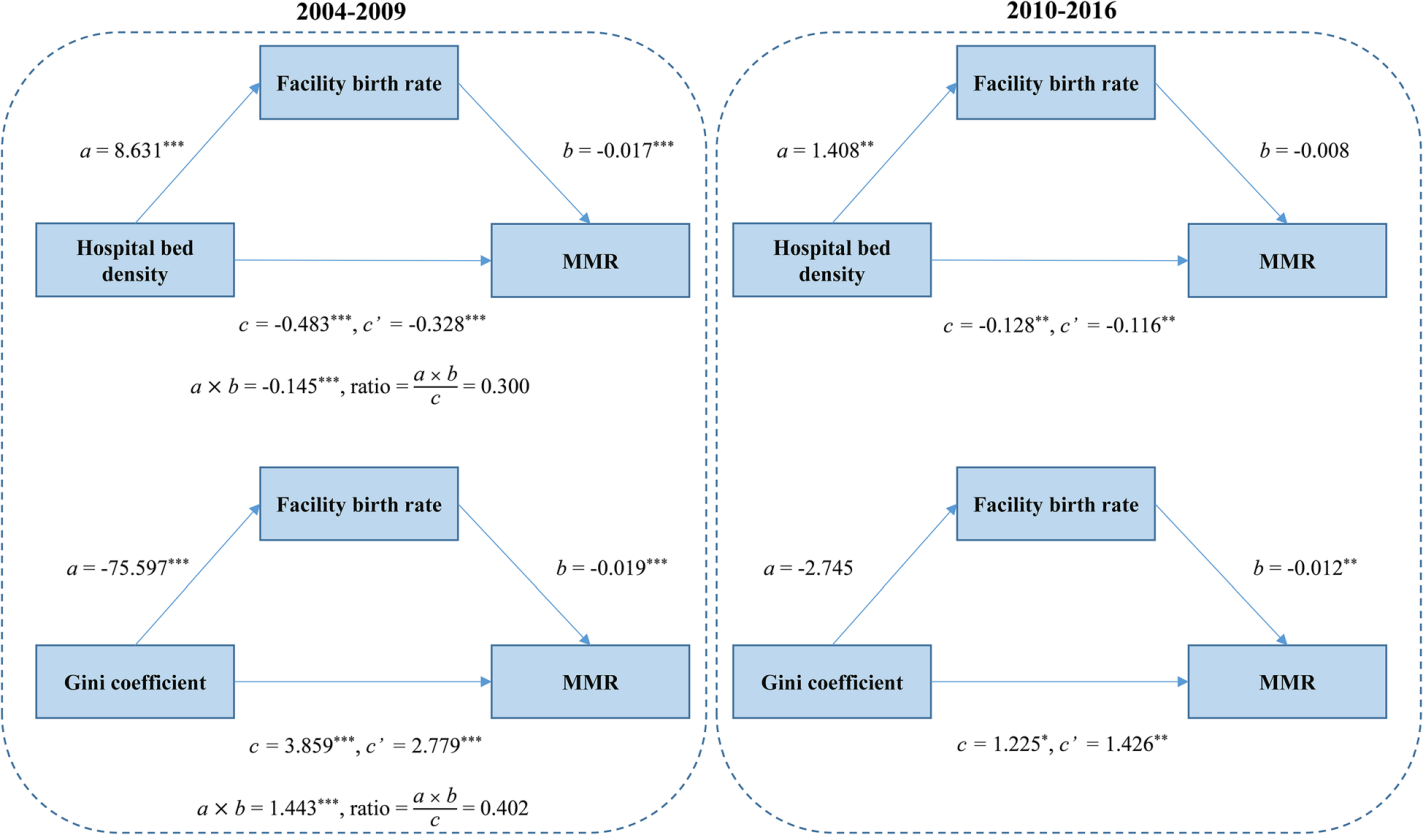
Mediation analysis of the effect of facility birth rate on hospital bed density or Gini coefficient and MMR. Note: *a*, *b*, *c* and *c*’ are regression coefficients. *a* × *b* denotes the indirect effects from hospital bed density (Gini coefficient) to MMR via facility birth rate. Ratio means the proportion of indirect effect accounting for the total effect. ^***^, ^**^ and ^*^ denote 1, 5 and 10% significance levels, respectively

The mediating influence of facility birth rate on the effect of hospital bed density or Gini coefficient on MMR was presented in Fig. 7. Between 2004 and 2009, hospital bed density was significantly associated with MMR (*c* = − 0.483, *P* < 0.001) and facility birth rate (*a* = 8.631, *P* < 0.001). There was still a significant negative association between hospital bed density and MMR after adjusting for facility birth rate (*c*’ = − 0.328, *P* < 0.001). Facility birth rate demonstrated a significantly negative association with MMR when the hospital bed density (*b* = − 0.017, *P* < 0.001) was adjusted. These estimation results above demonstrated a significant mediating effect from hospital bed density to MMR via facility birth rate (*a* × *b* = − 0.145, *P* < 0.001). The proportion of this indirect effect accounting for the total effect was 30%. Similar results could also be found for Gini coefficient and the proportion of the effect mediated by facility birth rate was 40.2%. In contrast, for the period from 2010 to 2016, facility birth rate showed an insignificant association with MMR when the hospital bed density (*b* = − 0.008, *P* > 0.05) was adjusted. It also found that Gini coefficient was insignificantly associated with facility birth rate (*a* = − 2.745, *P* > 0.05). These results revealed there were no mediating effects that could be identified during this period. The findings of mediation analysis indicated that hospital bed density and Gini coefficient have accelerated the penetration of facility births which consequently reduced the MMR, especially before the facility birth rate reached the summit in a universal range. When there was no potential for increasing facility births, the effect mediated by facility birth rate would be weaken.

## Discussion

Driven by the government’s strong commitment to the promotion of maternal health, MMR in China has experienced striking decline in all provinces between 2004 and 2016. Substantial investments in the overall healthcare system reform including increases in the hospital bed supply were considered to have accelerated such the progress. Based on the national statistical data in China between 2004 and 2016, this study depicted the time trends and regional variations in MMR, hospital bed density as well as the inequality of hospital bed distributions. In addition, the effects of hospital bed density as well as its distribution inequality on MMR were evaluated at the provincial level. Three major findings were reported as the result of our analysis. First, China has experienced considerable increase in nationwide hospital bed quantities between 2004 and 2016. Second, echoing the rise in hospital beds has been the steady improvement in distribution inequalities of hospital bed at the county level. Third, increased hospital bed supply as well as improved geographic distribution equity in hospital bed density were found to be significantly associated with reduced MMR, thus suggesting that the expansion of healthcare resource supply as well as the reduction in hospital bed distribution inequalities have the potential to produce measurable population health improvements.

As an essential component of healthcare resource, hospital bed density increased from 2.53 to 5.37 per 1000 population between 2004 and 2016. Such improvement might have largely benefited from the massive investments from both Chinese central and local governments aimed at enhancing hospital infrastructure constructions in a nationwide range [16, 43]. The governmental healthcare expenditure increased to 10.75 times in 2016 as compared with that in 2004. As reported by corresponding figures, such expenditure increased from 129.36 billion yuan in 2004 to 1391.03 billion yuan in 2016, thus reflecting a large expansion in governmental investments on nationwide healthcare promotion as part of the financial budgets [5]. In addition, great efforts has been put on strengthening the nationwide primary healthcare systems as well as promoting the development of private hospitals since the initiation of the latest phase of healthcare reform in 2009 [19, 44]. The rapid expansion of primary healthcare facilities and private hospitals reflected the expansion of nationwide healthcare resources. According to the latest National Plan of Health Care Service System (2015–2020), Chinese government has proposed an ambitious goal to enhance the development of hospital bed supply, denoting a specific target of achieving 6 hospital beds per 1000 population by 2020 [45]. In addition, the population’s raising demand on healthcare service might have served as a potent external driver force for stimulating the rapid growth of hospital bed supply [46]. Furthermore, there were substantial diversities in the geographic distribution of hospital bed density among different regions. When hospital bed density was calculated based on county population and areas, western regions in China were found to be particularly disadvantaged in average healthcare resource level compared with all the other regions. This finding was consistent with the previous literature which focused on the regional patterns of healthcare resource distributions in China [16, 47]. It should be noted that some western counties still demonstrated negative trends in hospital bed developments due to disproportionate reduction of hospital beds as well as the increase of general population size.

Our findings indicated that China has made significant progress in improving the geographic distribution inequalities of hospital beds at county levels between 2004 and 2016. Such achievement has been facilitated by a persistent focus addressed at governmental levels in order to ensure rational allocations of healthcare resources while promoting the equity and accessibility of obtaining healthcare services. In China, there were about 2000 rural counties that account for two thirds of the total county-level administrative units [17], thus such urban-rural gaps embedded in healthcare resource distribution might have been the leading contributor to healthcare resource distribution inequalities within each province [18]. Since the healthcare reform in 2009, rapid development of local healthcare systems has been witnessed in rural areas, with a substantial increase of hospital bed per 1000 population reported from 2.60 in 2010 to 3.91 in 2016 [5]. Specifically, the promotion of financial transfer payments has been adopted by the central government as a tactic for facilitating the development of healthcare systems in under-developed areas, especially in the western and central regions. Special funding programs aimed at healthcare facility constructions has also improved primary healthcare in rural areas [44]. Moreover, an educational program was launched in 2010 in order to increase the numbers of healthcare professionals in rural areas through attracting medical students to work in rural regions after graduation with their tuition exempted [48]. All these policies and strategies have largely narrowed such urban-rural gaps in healthcare resource allocations, which were essential to improving the overall healthcare resource inequalities within each province. Nevertheless, our results showed that Hainan, Beijing, Qinghai and Shanghai provinces still presented an upward trend of Gini coefficient between 2004 and 2016, based on which two potential scenarios would have to be considered. For highly developed areas with sufficient hospital beds, especially Beijing and Shanghai, such rising inequalities were more likely to be induced by an uneven expansion of hospital beds in certain regions. In contrast, such widened gaps in Qinghai and Hainan which are featured by vast land areas as well as under-developed economic status, tend to be attributed to their under-developed regional healthcare systems. Therefore, it should be highlighted that in such under-developed regions, the expansion of hospital bed supply should be proposed as the key strategy for meeting the basic demands of healthcare services instead of improving the equity of healthcare resource distribution in these regions based on the fairly low level of regional hospital bed quantities. Furthermore, it was noteworthy that an extreme inequality of healthcare resource distribution was found in Tibet which constantly remained lagging behind all the other provinces. Such findings suggested that the improvement of healthcare resource allocation in a nationwide range should be addressed as a long-term strategy, especially in economically disadvantaged areas.

In this study, hospital bed density as well as its inequality in geographic distributions demonstrated a significant association with MMR. This finding was consistent with our hypothesis and provided empirical evidence that the expansion of hospital bed supply as well as improving the distribution inequality of healthcare resources have contributed to the nationwide MMR reductions. In particular, hospital bed density was proven to be a powerful indicator in our model, with a raise of 1 hospital bed per 1000 population yielding 10.60% decline of MMR on average. Several studies have confirmed that higher hospital bed density was persistently associated with lower MMR [49–51]. As the supply-side input of healthcare systems, the number of hospital beds partly reflects the hospital volume and capability and ensures the availability of maternal healthcare services, such as in-hospital delivery services. More importantly, the improvement in hospital bed distribution inequalities should be highlighted as another key contributor to inducing the decline in MMR. The narrowed gap in hospital bed distributions at county levels was found to be crucial for reducing the inequality in obtaining access to healthcare services [17]. Under such circumstances, it is not difficult to predict that under-developed counties would have more potential for utilizing maternal healthcare services thus obtaining more benefits from MMR reductions. As part of our results, facility birth rate was found to have played a mediating role in the association of hospital bed density or Gini coefficient with MMR, especially during the period between 2004 and 2009. Such significant mediating effect suggested that the expansion of hospital beds as well as the improvement of hospital bed distribution inequalities have substantially facilitated the penetration of in-hospital births, and ultimately achieved in the reduction of MMR. This encouraging finding provided three important implications. First, it confirmed that healthcare outcome disparities could be largely attributed to the inequality in healthcare resource distributions at the aggregate level. In attempt to mitigate such healthcare outcome disparities in a nationwide range, constant efforts would have to be made at central governmental levels in order to facilitate rational distributions of healthcare resources, with exceptional emphasis posed on propelling the development of primary healthcare systems. Second, our findings highlighted the remarkable achievements in the enhancement of nationwide healthcare equity and accessibility as the result of long-term efforts made in this aspect at the central governmental level. Third, our findings provided valuable lessons for other developing countries confronted with the same goal of achieving equity in healthcare resource allocations. In spite of our valuable findings, it should be noted that the substantial reduction in hospital bed allocation inequalities between 2004 and 2016 merely reflected the general trends of hospital bed distributions, which failed to further provide evidences on whether an equity in healthcare quality could be achieved among various regions as the result of evenly allocated hospital bed distributions, thus leaving this question to be further investigated which was more directly reflective of population health status. Therefore, accurate information about the equity of healthcare quality should be considered in future processes of assessing the effects of healthcare resource disparities on health outcome inequities.

### Limitations

This study has several limitations that should be noted. First, in this study hospital beds was adopted as a proxy indicator for reflecting healthcare resources while neglecting other essential components as part of healthcare resources such as healthcare workforce and financing. However, in a healthcare system, healthcare infrastructures, healthcare workforce as well as financing were typically found to be highly correlated. Previous studies have identified a similar pattern of geographic distribution inequalities in the aspect of healthcare workforce as what we found for hospital beds in this study. Based on these knowledge, hospital beds should still be considered as a reasonable indicator for reflecting the distribution of healthcare resources. Secondly, due to the data availability, we failed to include some important factors that may confound the association between hospital bed capacity as well as its inequality and MMR, such as the health seeking behaviors of pregnant women and government subsidies for women to delivery at health facilities. These valuable indicators need to be further considered in the future analysis. Thirdly, we are limited to address the potential simultaneity bias where the development of hospital beds could also be affected by the population health (e.g., MMR). If there was a positive effect of MMR on hospital beds as well as a negative effect of hospital beds on MMR, we would see an upward bias in the estimated coefficient of hospital beds. Finally, our study failed to incorporate a list of indicators reflective of healthcare quality due to data availability issue, thus we were only able to evaluate the distribution of healthcare resources from the perspective of healthcare resource quantities. Therefore, it is highly recommended that detailed information about healthcare quality be collected and adopted in future studies in order to conduct comprehensive evaluations on the impact of healthcare resource distribution disparities in terms of affecting healthcare outcomes among different regions [18].

## Conclusions

China has made striking success in reducing MMR between 2004 and 2016. This study provided empirical evidences indicating that this substantial reduction could be attributed to the hospital bed supply as well as its geographic distribution inequality at the provincial level. Specifically, regions with higher hospital bed density and more evenly allocated hospital bed distributions demonstrated greater potential for achieving a much lower MMR. Our findings highlighted the inequalities in healthcare resource distributions as an essential contributor to inducing varied healthcare outcomes among different regions, which were expected to provide evidence-based implications for future policy-making procedures in order to optimize healthcare resource allocations in a nationwide range, thus ultimately promoting the equity and accessibility to obtaining healthcare services. Despite such considerable progress, it is noteworthy that substantial inequalities still remain in China, especially among the western regions. Under such circumstances, constant efforts should be made at governmental levels in order to effectively improve healthcare resource allocations in economically disadvantaged regions, which might include the provision of special funding programs aimed at the improvement of regional healthcare systems in under-developed regions, as well as the provision of stronger incentives for attracting healthcare professionals to work in indigent and distant counties. Our findings were also expected to provide valuable lessons for other developing countries with the same aim of promoting the equity in healthcare resource allocations, for which a critical priority needs to be addressed on improving healthcare resource distributions among different regions in order to achieve the penetration of universal healthcare coverage.

## Supplementary Information

**Additional file 1: Appendix Table 1**. Description of included variables. **Appendix Table 2**. Absolute differences in maternal mortality ratio by income level of provinces in China, 2004–2016. **Appendix Table 3**. Changes in geographic distribution of hospital beds by county, China, 2004–2016. **Appendix Table 4**. Correlation matrix of included independent variables. **Appendix Table 5**. Collinearity diagnostics of included independent variables.

## Data Availability

The datasets used during the current study are available from the corresponding author on reasonable request.
