# Supplementary material for: Hospital bed supply and inequality as determinants of maternal mortality in China between 2004 and 2016
Source: Int J Equity Health. 2021 Jan 30;20:51. doi: 10.1186/s12939-021-01391-9 (PMC7846917; doi:10.1186/s12939-021-01391-9)
Supplement: Supplementary file 1 — Additional file 1: Appendix Table 1. Description of included variables. Appendix Table 2. Absolute differences in maternal mortality ratio by income level of provinces in China, 2004–2016. Appendix Table 3. Changes in geographic distribution of hospital beds by county, China, 2004–2016. Appendix Table 4. Correlation matrix of included independent variables. Appendix Table 5. Collinearity diagnostics of included independent variables. [file 12939_2021_1391_MOESM1_ESM.docx]

#### Appendix Table 1 Description of included variables

| **Indicator** | **Resource** | **Durations** | **Measurement** | **Units** | **Sampling techniques** |
| --- | --- | --- | --- | --- | --- |
| Hospital beds per 1,000 population | National Health Statistical Yearbooks | 2004-2016 | Annual number of hospital beds (including all healthcare institutions except for clinics) per 1,000 population for 31 provinces. | Beds per 1,000 population | Healthcare institutions or health administrative institutions were required to regularly report this indicator by a national reporting system to National Health Commission in China. |
| Gini coefficient calculated by hospital beds per 1,000 population | China City Social and Economic Statistical Yearbooks | 2004-2016 | Annual number of hospital beds (only including hospitals and health centers) per 1,000 population for 265 districts. | Beds per 1,000 population | Healthcare institutions or health administrative institutions were required to regularly report this indicator by a national reporting system to National Health Commission in China. |
|  | China County Social and Economic Statistical Yearbooks |  | Annual number of hospital beds (including all healthcare institutions except for clinics) per 1,000 population for 2,082 counties. |  | Healthcare institutions or health administrative institutions were required to regularly report this indicator by a national reporting system to National Health Commission in China. |
| MMR | National Health Statistical Yearbooks | 2004-2006 | Annual number of maternal deaths per 100,000 live births. | Deaths per 100,000 live births | The surveillance data were collected from 116 randomly selected monitoring sites (328 in total) in 31 provinces. |
|  |  | 2007-2016 |  |  | The surveillance data were collected from 116 randomly selected monitoring sites (336 in total) in 31 provinces. |
| Facility birth rate | National Health Statistical Yearbooks | 2004-2016 | Ratio of annual number of live births in township hospital or above this level to all live births. | Per hundred | 31 provinces were required to yearly report this indicator to National Health Commission in China. |
| Birth rate | National Statistical Yearbooks | 2004, 2006-2009, 2011-2014, 2016 | Ratio of annual number of live births to average population size in the same period. | Per thousand | The survey were conducted by a stratified multi-stage sampling method covering 1‰ population in China. |
|  |  | 2005, 2015 |  |  | The survey were conducted by a stratified multi-stage sampling method covering 1% population in China. |
|  |  | 2010 |  |  | Census data. |
| Female illiteracy | National Statistical Yearbooks | 2004, 2006-2009, 2011-2014, 2016 | Ratio of women who were illiterate in the population aged 15 years or older. | Per hundred | The survey were conducted by a stratified multi-stage sampling method covering 1‰ population in China. |
|  |  | 2005, 2015 |  |  | The survey were conducted by a stratified multi-stage sampling method covering 1% population in China. |
|  |  | 2010 |  |  | Census data. |
| GDP per capita | National Statistical Yearbooks | 2004, 2006-2009, 2011-2014, 2016 | Ratio of Gross Domestic Product to population size. | Yuan per capita | The population size were obtained by a stratified multi-stage sampling survey covering 1‰ population in China. |
|  |  | 2005, 2015 |  |  | The population size were obtained by a stratified multi-stage sampling survey covering 1% population in China. |
|  |  | 2010 |  |  | The population size were obtained by census. |
| Urbanization rate | National Statistical Yearbooks | 2004, 2006-2009, 2011-2014, 2016 | Ratio of urban population to general population. | Per hundred | The population size were obtained by a stratified multi-stage sampling survey covering 1‰ population in China. |
|  |  | 2005, 2015 |  |  | The population size were obtained by a stratified multi-stage sampling survey covering 1% population in China. |
|  |  | 2010 |  |  | The population size were obtained by census. |

#### Appendix Table 2 Absolute differences in maternal mortality ratio by income level of provinces in China, 2004-2016

|  | **Maternal mortality ratio (per 100,000 live births)** | | | |
| --- | --- | --- | --- | --- |
|  | **2004** | **2008** | **2012** | **2016** |
| **Region** |  |  |  |  |
| Highest income provinces | 18.74 | 12.22 | 5.18 | 5.69 |
| Upper middle-income provinces | 26.09 | 15.36 | 10.77 | 9.40 |
| Middle income provinces | 61.29 | 26.15 | 14.65 | 13.06 |
| Lower middle-income provinces | 51.95 | 34.44 | 14.71 | 13.40 |
| Lowest income provinces | 63.69 | 32.68 | 20.10 | 17.53 |
| **Absolute difference in MMR (95% *CI*)** |  |  |  |  |
| Upper middle minus highest income provinces | 7.39 (4.58-10.20) | 3.14 (1.14-5.13) | 5.62 (4.11-7.13) | 3.72 (2.51-4.94) |
| Middle minus highest income provinces | 42.61 (38.87-46.34) | 13.91 (11.57-16.26) | 9.50 (7.72-11.28) | 7.38 (5.58-9.18) |
| Lower middle minus highest income provinces | 33.26 (29.26-37.26) | 22.21 (19.31-25.12) | 9.55 (7.93-11.17) | 7.74 (6.39-9.08) |
| Lowest minus highest income provinces | 44.98 (41.31-48.65) | 20.47 (18.04-22.91) | 14.96 (13.13-16.78) | 11.86 (10.26-13.46) |

**Note:** MMRs: maternal mortality ratios, *CI*: confidence interval.

#### Appendix Table 3 Changes in geographic distribution of hospital beds by county, China, 2004-2016

|  | **Hospital beds per 1000 population, Mean (95% *CI*)** | | |  | **Hospital beds per 1000 population per 1 km^2^, Mean (95% *CI*)** | | |
| --- | --- | --- | --- | --- | --- | --- | --- |
|  | **2004** | **2016** | **Within-county change, 2004 to 2016** |  | **2004** | **2016** | **Within-county change, 2004 to 2016** |
| **Total** | 2.28  (2.21-2.34) | 4.54  (4.45-4.64) | 2.27  (2.20-2.33) |  | 0.0020  (0.0018-0.0022) | 0.0033  (0.0030-0.0035) | 0.0012  (0.0010-0.0014) |
| **Region** |  |  |  |  |  |  |  |
| Eastern region | 2.32  (2.21-2.43) | 4.37  (4.21-4.52) | 2.05  (1.95-2.14) |  | 0.0028  (0.0023-0.0033) | 0.0042  (0.0036-0.0048) | 0.0014  (0.0011-0.0018) |
| Central region | 2.28  (2.16-2.40) | 4.50  (4.32-4.67) | 2.22  (2.11-2.33) |  | 0.0025  (0.0020-0.0030) | 0.0036  (0.0032-0.0041) | 0.0011  (0.0007-0.0015) |
| Western region | 2.24  (2.14-2.35) | 4.69  (4.54-4.85) | 2.45  (2.34-2.56) |  | 0.0012  (0.0010-0.0013) | 0.0023  (0.0020-0.0026) | 0.0011  (0.0010-0.0013) |

**Note:** *CI*: confidence interval.

#### Appendix Table 4 Correlation matrix of included independent variables

| **Variables** | **Hospital beds per 1000 population** | **Gini coefficient** | **Birth rate, ‰** | **Female illiteracy, %** | **Log of GDP per capita** | **Urbanization rate, %** |
| --- | --- | --- | --- | --- | --- | --- |
| **Hospital beds per 1000 population** | 1 |  |  |  |  |  |
| **Gini coefficient** | -0.452^***^ | 1 |  |  |  |  |
| **Birth rate, ‰** | -0.244^***^ | 0.467^***^ | 1 |  |  |  |
| **Female illiteracy, %** | -0.453^***^ | 0.456^***^ | 0.506^***^ | 1 |  |  |
| **Log of GDP per capita** | 0.690^***^ | -0.644^***^ | -0.454^***^ | -0.554^***^ | 1 |  |
| **Urbanization rate, %** | 0.543^***^ | -0.733^***^ | -0.576^***^ | -0.593^***^ | 0.876^***^ | 1 |

**Note:** ^***^, ^**^ and ^*^ denote 1, 5 and 10 % significance levels, respectively.

#### Appendix Table 5 Collinearity diagnostics of included independent variables

| **Variables** | ***VIF*** | |
| --- | --- | --- |
|  | **Model 1**  **(All variables)** | **Model 2**  **(Excluding**  **urbanization rate)** |
| **Hospital beds per 1000 population** | 2.068 | 1.987 |
| **Gini coefficient** | 2.200 | 1.839 |
| **Birth rate, ‰** | 1.662 | 1.540 |
| **Female illiteracy, %** | 1.735 | 1.690 |
| **Log of GDP per capita** | 5.997 | 2.861 |
| **Urbanization rate, %** | 6.519 |  |

**Note:** *VIF*: variance inflation factor.
